# Altered intrinsic brain activity associated with outcome in frontal lobe epilepsy

**DOI:** 10.1038/s41598-019-45413-7

**Published:** 2019-06-20

**Authors:** Xintong Wu, Wenyu Liu, Weina Wang, Hui Gao, Nanya Hao, Qiang Yue, Qiyong Gong, Dong Zhou

**Affiliations:** 1 Departments of Neurology, Huaxi MR Research Center (HMRRC), West China Hospital, Sichuan University, No. 37 GuoXue Alley, Chengdu, 610041 China; 2 Departments of Radiology, West China Hospital, Sichuan University, No. 37 GuoXue Alley, Chengdu, 610041 China

**Keywords:** Epilepsy, Epilepsy

## Abstract

Frontal lobe epilepsy (FLE) is the second most common type of the focal epilepsies. Our understanding of this disease has been revolutionized over the past decade, but variable treatment outcomes persist and the underlying functional mechanisms responsible for this have yet to be deciphered. This study was designed to determine how intrinsic brain connectivity related to treatment response in patients with FLE. 50 patients with FLE and 28 healthy controls were enrolled in this study and underwent functional MRI at baseline. At the end of 12-month follow up period, all patients with FLE were classified, based on their responses to AEDs treatment, into drug-responsive and drug-refractory groups. The amplitude of low-frequency fluctuation (ALFF) was calculated amongst the three groups in order to detect regional neural function integration. The responsive group showed decreased ALFF only in the left ventromedial prefrontal cortex (vmPFC), while the refractory group showed decreased ALFF in the left vmPFC, right superior frontal gyrus (SFG), and supramarginal gyrus (SMG) relative to healthy controls. In addition, both the responsive and refractory groups showed increased ALFF in the precuneus and postcentral gyrus when compared to the healthy controls. Furthermore, the refractory group exhibited significantly decreased ALFF in the left vmPFC, right SFG and SMG, relative to the responsive group. Focal spontaneous activity, as assessed by ALFF, was associated with response to antiepileptic treatment in patients with FLE. Patients with refractory frontal lobe epilepsy exhibited decreased intrinsic brain activity. Our findings provide novel neuroimaging evidence into the mechanisms of medically-intractable FLE at the brain level.

## Introduction

Affecting about 50 million people worldwide, epilepsy has in recent years been recognized as a serious public health concern^1^. More than 30% of patients with epilepsy continue to experience seizures despite adequate treatment with antiepileptic therapy^2^. Approximately 25% of all cases of refractory focal epilepsy are categorized as frontal lobe epilepsy (FLE). Abnormal neuronal connections are increasingly postulated as a crucial factor in the pathogenesis of epilepsy^3^.

Following temporal lobe epilepsy (TLE), FLE has long been considered the second most prevalent type of the focal epilepsies^4^. In FLE, seizures originate in the frontal lobe, but the clinical symptoms of these seizures are variable and dependent on the brain regions involved or the functional networks impacted^5^, although most are recognized as multi-cognitive defects and motor-related abnormality networks^6^. Additionally, the interictal discharges arising from a unilateral focus in frontal lobe are more likely to spread to both hemispheres and result in secondary bilateral synchrony^7^. These phenomena implicate abnormality in multiple functional systems in FLE patients.

The clinical outcome of FLE is also widely variable. It is reported that, of all patients whose long-term seizure-free outcome has been reported, those with isolated FLE represent approximately 11%^8^. There is also considerable heterogeneity in seizure response and little data are available to identify patients with FLE who may benefit from treatment. Furthermore, this individual variability extends to the brain networks responsible for FLE. The previously identified epileptic network pathways for FLE are broad but include abnormal functioning in focal regions of the thalamus, frontal cortex, precuneus, insula, and limbic regions^9–12^, with variation reported between patients. However, analysis of the regional cerebral function in FLE has not been well investigated. More importantly, there is also a lack of evidence to associate seizure outcome with resting-state neural activity in this population.

To investigate the dysfunction of intrinsic regional activity, the amplitude of spontaneous brain oscillations was measured as amplitude of low-frequency fluctuations (ALFF)^13^. The present investigation assessed the regional brain activity in patients with FLE used resting-state fMRI and compared them to those of healthy controls, and also brought forth and tested the hypothesis that intrinsic connectivity is related to treatment response by dividing FLE patients into refractory and responsive groups. Additionally, the associations between regional activity values and epilepsy duration were examined. This will further characterize brain connections in a larger sample of patients with FLE in an effort to provide deep insights into the clinical implications and resulting medical interventions utilized in FLE treatment, and may also contribute to better evaluating seizure prognosis.

## Results

### Subject characteristics

Fifty patients with FLE (24 females and 26 males) and 28 healthy controls were enrolled in this study and underwent functional MRI at baseline. At the end of 12-month follow up period, all patients with FLE were classified, based on their responses to AEDs treatment, into drug-responsive and drug-refractory groups. The age of the patients ranged from 13 to 63 years with a mean of 29.2 years (standard deviation [SD]: 11.6 years). The mean duration of epilepsy was 10.1 years (SD: 7.3 years). The neurologic and mental examination results were normal in all patients. They were all right-handed. There was no difference in gender and age among the three groups (P > 0.05). All of the patients with FLE were on at most two antiepileptic drugs and they had no seizure for at least one month before fMRI scanning. Clinical information including medications, seizure types, seizure frequency, MMSE scores, EEG results, and PET results were shown in Supplementary Table 1.

### Subgroup characteristics

Patients were classified into either the “refractory” group or the “responsive” group based on their responses to AEDs treatment at the end of 12-month follow up period.

During the follow up period, most of the patients (44/50) did not change the medication or the dose. However, the medication was adjusted in six patients, either by increasing the dose of AEDs or adding a new AED (from monotherapy to polytherapy) during the follow up period. All of the six patients turned out to be in the refractory group. Patients in the responsive group did not change the medication during 12-month follow up period. In the refractory group, most patients (15/25, 60%) continued to have high-frequency seizures (at least 6 seizures/year) during the course of the disease despite appropriate treatment with AEDs. In the responsive group, following treatment with AEDs, seizures were reduced by >50%, and patients usually maintained a low frequency of seizures. Monthly or daily seizures were never observed in this subgroup.

### Clinical comparison between the responsive and refractory groups

Both the responsive and the refractory groups exhibited no significant differences in age of onset, duration, treatment time, follow-up time, seizure type, existence of aura, family history, years of education, and risk factors (P > 0.05). The baseline clinical and demographic information for the participants is detailed in Table 1.

**Table 1:** Demographic information of refractory and responsive patients with FLE, and HCs at baseline.

Characteristics	Refractory Patients (n = 25)	Responsive Patients (n = 25)	HCs (n = 28)	P Value
Age (years)	27.8 ± 8.9	30.7 ± 13.8	29.36 ± 10.26	0.671^a^
Sex (male/female)	12/13	14/11	14/14	0.571^b^
Education (years)	8.6 ± 3.2	9.1 ± 3.6	10.3 ± 2.9	0.146^a^
Age at onset (years)	16.1 ± 8.7	22.3 ± 14.6	—	0.072^c^
Duration (years)	11.8 ± 8.9	8.3 ± 4.8	—	0.098^c^
Family history (+/−)	1/24	0/25	—	0.312^b^
Aura (+/−)	3/22	4/21	—	0.684^b^
Seizure type (focal-only/SGTCS)	4/21	2/23	—	0.544^b^
Sleep-related hypermotor epilepsy	10/15	11/14	—	0.775^b^
Previously treated/naïve	25/0	25/0	—	NA
Medication (monotherapy/polytherapy)	6/19	8/17	—	0.528^b^
Seizure frequency at baseline (/year)	9.44 ± 4.16	8.92 ± 3.99	—	0.654^c^
Seizure frequency after follow up (/year)	7.81 ± 3.68	3.40 ± 1.96	—	<0.001^c^

Values are mean ± SD.

^a^One-way analysis of variance.

^b^Chi-square test.

^c^Two-tailed two-sample *t* test.

### ALFF group differences

**Figure 1:**
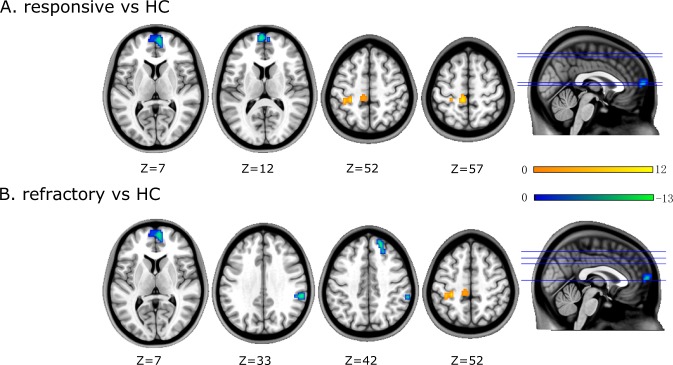
Brain regions showing significant differences in ALFF between refractory or responsive FLE and controls. Warm and cold colors indicate regions with increased and decreased ALFF values, respectively. At right, color bars indicate T values from global voxel-based post-hoc analysis. Further details are presented in Table 2.

When compared to the healthy controls, the responsive group displayed a significant decrease in ALFF in the left ventromedial prefrontal cortex (vmPFC), whereas the refractory group showed decreased ALFF in the left vmPFC, right superior frontal gyrus (SFG), and supramarginal gyrus (SMG). In addition, both the responsive and refractory groups showed an increase in ALFF in the precuneus and postcentral gyrus, in comparison to the healthy controls (Fig. 1). The information for the peak coordinates, the P values, and the brain regions is detailed in Table 2.

**Table 2:** Significant Differences of ALFF between refractory or responsive Patients with FLE and HCS.

Brain region	voxels	Peak MNI coordinates			P value	T-value
		X	Y	Z		
**responsive** < **HCs**						
vmPFC_L	75	−6	63	12	0.005	2.899
**responsive** > **HCs**						
Postcentral gyrus_L	25	−30	−33	52	<0.0001	4.882
Precuneus gyrus_L	21	−9	−36	60	<0.0001	4.427
r**efractory < HCs**						
vmPFC_L	75	−6	63	12	<0.0001	6.124
SupraMarginal gyrus _R	36	60	−36	33	<0.0001	5.369
Superior frontal gyrus_R	27	18	45	42	<0.0001	5.718
**refractory** > **HCs**						
Postcentral gyrus_L	25	−30	−33	52	<0.0001	5.395
Precuneus gyrus_L	21	−9	−36	60	<0.0001	4.156

Abbreviations:

ALFF: Amplitude of low-frequency fluctuation; FLE: Frontal lobe epilepsy;

HCS: Healthy controls; MNI, Montreal Neurological Institute; L, left; R, right;

vmPFC, ventromedial prefrontal cortex.

**Figure 2:**
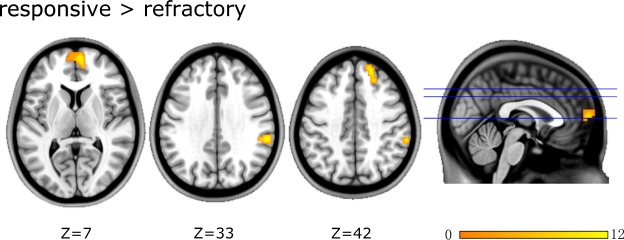
Brain regions showing significant differences in ALFF between refractory and responsive FLE. Warm colors indicate regions with increased ALFF values. At right, color bars indicate T values from global voxel-based post-hoc analysis. Further details of these regions are presented in Table 3.

More important was the direct comparison, where the refractory group exhibited decreased ALFF in the left ventromedial prefrontal cortex (vmPFC), right superior frontal gyrus (SFG), and supramarginal gyrus (SMG) relative to the responsive group (Fig. 2). There was no increase in the ALFF in the brain regions of the refractory group compared to the responsive group. The information for the peak coordinates, the P values and the brain regions is detailed in Table 3.

**Table 3:** Significant differences of ALFF between refractory patients and responsive patients with FLE.

Brain region	voxels	Peak MNI coordinates			P value	T-value
		X	Y	Z		
**Regions with decreased ALFF in “refractory” patients relative to “responsive” patients**						
vmPFC_L	75	−6	63	12	0.007	2.814
SupraMarginal gyrus_R	36	60	−36	33	<0.0001	4.601
Superior frontal gyrus_R	27	18	45	42	0.0002	3.993

Abbreviations:

ALFF: Amplitude of low-frequency fluctuation; FLE: Frontal lobe epilepsy;

MNI, Montreal Neurological Institute; L, left; R, right;

vmPFC, ventromedial prefrontal cortex.

### Correlations between ALFF and duration of epilepsy

**Figure 3:**
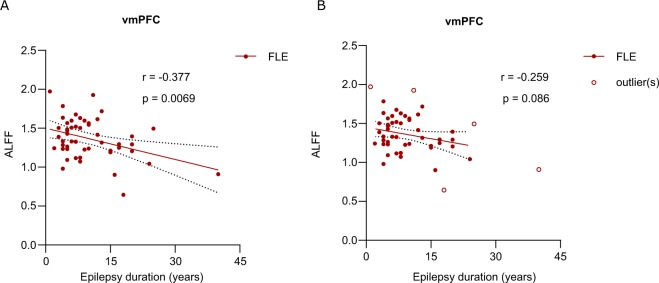
Correlation between ALFF and epilepsy duration in patients with FLE. (**A**) ALFF values in the vmPFC was negatively correlated with epilepsy duration (P < 0.05). The solid line and dashed lines represent the best-fit line and 95% confidence interval of Pearson correlation. (**B**). After the exclusion of five outliers, no significant correlations were identified between epilepsy duration and ALFF changes in the vmPFC (r = −0.259, p = 0.086).

Linear Pearson correlation coefficients between altered regional ALFF values and epilepsy duration in FLE patients were calculated, and the results showed that a correlation was only detected between ALFF in the vmPFC and epilepsy duration (r = −0.377, p = 0.007) (Fig. 3A). ALFF in the other brain regions of FLE patients were not correlated with the duration epilepsy. A sensitivity sub-analysis was also performed. After the exclusion of outliers, no significant correlations were identified between epilepsy duration and ALFF changes in the vmPFC (r = −0.259, p = 0.086) (Fig. 3B). Furthermore, we did not detect a correlation between the ALFF values and epilepsy duration in either the refractory group or the responsive group.

## Discussion

In the present study, we used ALFF to examine medically-responsive and refractory patients with FLE to demonstrate specific alteration patterns, revealing that varying responses to antiepileptic drugs may be attributed to different patterns of intrinsic regional brain dysfunction. These findings suggest that an abnormal substrate in the brain might be characteristically responsible for refractory FLE; this information could be paramount to gaining insights into the pathophysiological mechanisms of the disease and providing different therapeutic approaches at the brain level.

Previous studies have supported the finding of abnormalities in ALFF measurements of local brain activity in patients with FLE^14^, which are thought to potentially result from seizure-induced damage to the epileptic brain. In our study, the responsive group presented with decreased ALFF in only the left vmPFC, while the refractory group presented with decreased ALFF in the left vmPFC, right SFG, and SMG. Additionally, both the responsive and refractory groups showed increased ALFF in the precuneus and postcentral gyrus, when compared to the HCs.

We observed significant decreased ALFF of the vmPFC, an important hub of default mode network (DMN), closely connected to the limbic system^15^. Generally, DMN has been described as abnormal in epilepsy patients^16^. There is a known association between frontal lobe dysfunction and behavioral impairment. Numerous previously reported clinical studies have described the association between social or behavior traits and the alteration of vmPFC^17,18^. The decreased ALFF of vmPFC that we observed in the present study indicated a more specific, epilepsy–affective interaction, which could account for the behavioral and emotional comorbidities in patients with FLE. Previous studies have shown that in patients with TLE, DMN abnormalities were caused by interictal discharges or chronic seizures^19^, which might result in the functional impairment. We can assume that underlying activity in these crucial hubs possibly become more dysfunctional in patients with chronic FLE. However, in the current study, vmPFC amplitude values were not found to be associated with duration of epilepsy when outliers were excluded.

Impairment of consciousness is a key feature of FLE in clinical practice^20^. The integrity of the precuneus is required for the maintenance of consciousness, and it has been reported that, in adults and children with FLE, involvement of the precuneus is largely consistent with the impairment of DMN^12,14^. We detected altered amplitude values in the precuneus and superior frontal gyrus, suggesting that, in FLE, DMN regions may play an important role in disrupting the normal integrative functions of consciousness. Additionally, it has been reported that patients with FLE^14^, TLE^19^ and GTCS^21^ exhibit decreased connectivity within the perceptual network including sensorimotor network (SMN), which is comprised of the primary motor cortex in the precentral gyrus, the primary sensory cortex in the postcentral gyrus, and the posterior aspect of the superior frontal gyrus^22^. Because repeated seizure activity generates network disturbances^23^, it is possible that these findings may be responsible for the sensorimotor deficits observed in FLE.

Of patients referred to tertiary care centers for pharmacoresistant focal epilepsy, approximately 18% have FLE^24^. Additionally, approximately 11% of all patients whose long-term seizure-free outcome has been reported have isolated FLE^25^. However, the underlying functional mechanism responsible for the variation in treatment outcomes remains unknown. We performed a direct comparison between the groups of patients with refractory and responsive FLE, and also revealed striking differences between them. Specifically, relative to the responsive group, the refractory group exhibited a decrease in ALFF in the left vmPFC, right SMG, and SFG, whereas no brain regions with increased ALFF were found. Because the demographic, duration, treatment time, and follow-up time data between the two groups were so well matched, the results of the direct comparison could be regarded as representing intrinsic brain activity related to refractory FLE itself.

When examining the correlation between brain activity and treatment response, it is important to note that previous neuroimaging evidence suggests that post-therapy seizure reduction is linearly correlated with enhanced functional connectivity between the amygdala and prefrontal cortex^26^. Increasing evidence supports the identification of vmPFC as an essential component of the default mode network and its involvement in limbic areas. Persistent disruption in the bilateral vmPFC produced by frequent seizures, led to reversible interictal antisocial behavioral disorders^27^. Furthermore, patients with FLE exhibited decreased connectivity within the motor network, correlating with the number of lifetime seizures^28^. This result demonstrated a potential relationship between seizure activity and changes in motor network organization. Our current study revealed altered ALFF measurements the SMG and SFG in epileptic patients with FLE. The implication of this finding could be quite impactful because the SMG is responsible for receptive language function and also involved in the sensation and perception of various stimuli and the SFG is notably associated with executive control and attention regulation^29^. Therefore, the intrinsic function abnormalities in these brain regions could not only result in sensorimotor deficits in FLE, but may reveal further dysfunctional intrinsic activity as frontal lobe seizures become more frequent and intractable.

Furthermore, accumulating studies indicate that FLE is a diffuse network disorder that affects the structure, function, and metabolism of the whole brain. Consistent with our results, another rs-fMRI study^10^ using dynamic functional network connectivity (dFNC) analysis also found that FLE patients exhibited decreased dFNC in almost all patterns, suggesting a disturbed communication between the frontoparietal system and other systems. A topology study has found altered node degree, clustering coefficient and local efficiency in basal ganglia and limbic system by graph theoretical analyses^30^. Moreover, other studies have found abnormal functional connectivity within and across resting-state networks, with less interconnectivity in subnetworks^31^. Similar to the reconfiguration of these functional connectomes, evidence from diffusion weighted MRI revealed that an increase in structural modularity accompanied stronger cognitive impairment^30^.

This study did have several limitations. First, there are many different types of frontal lobe epilepsy depending on the location of the epileptogenic region, and the underlying cause. We included MRI-negative FLE patients, based on typical clinical symptoms and results of long-term video EEG. Secondly, the important information were neglected when factoring or analysis, including the dose and type of AEDs. Data the EEG was not obtained during the resting-state fMRI and data of cardiac and respiratory fluctuations were also not obtained. A limited number of participants and choices of non-stringent thresholds were recruited in the current study, which decreased the statistical power. A larger sample of newly diagnosed FLE patients and prospective design would be important for validation of our current findings. Finally, although our findings hinted at a possible shared intrinsic network between the refractory and responsive groups, the pathophysiological mechanism of medically intractable FLE remains controversial, thereby warranting follow-up analysis of the subjects included in the present study.

## Materials and Methods

### Subject criteria

Fifty patients with FLE were enrolled from the epilepsy center of West China Hospital in Chengdu. Initially, we totally recruited 76 patients with FLE in this study from outpatient clinic, epilepsy monitoring unit and inpatient ward. We obtained the demographic information, clinical data and fMRI image of each patient at the beginning. However, six patients were lost to follow up during 12-month period; five patients were excluded due to low quality of functional imaging data; nine patients had poor compliance and had changed the medication themselves or in other hospital, and other six patients were excluded because of baseline matching, according to inclusion criteria. Eventually, only 50 patients (and subsequently the 25/25) were enrolled for analysis. This study was approved by the local ethical committee of the West China Hospital of Sichuan University, and all subjects have provided informed consent. All methods were performed in accordance with the relevant guidelines and regulations.

Patients with FLE were diagnosed based on the International League against Epilepsy (ILAE) classification^32^, and inclusion criteria were as follows: (1) clinically confirmed cryptogenic localization-related epilepsy with an epileptic focus in the frontal lobe; (2) clinical presentation of one or more typical symptoms of FLE, including unilateral clonic seizures, tonic asymmetric seizures, or hypermotor seizures, usually short and tend to occur during sleep; (3) neurophysiological monitoring revealing ictal or interictal epileptic discharges during video-EEG; and (4) non-lesional structural MR imaging. During the inclusion period, the baseline characteristics were well balanced in this cohort. Those who used one or two antiepileptic drugs, and did not differ in seizure type or seizure frequency were enrolled in this study. The exclusion criteria were as follows: (1) brain malformations or prior brain surgery; (2) MRI incompatibility; (3) psychogenic non-epileptic seizures; (4) had seizures in last one month; (5) lost to follow up or incomplete clinical data.

Detailed clinical information including primary symptoms, age of seizure onset, duration of epilepsy, antiepileptic drugs (AEDs), and EEG profiles were collected using standardized questionnaires. An examination of mental state was measured by MMSE. The Annett Handedness Scale was used to measure the handedness of subjects. The response to AEDs was closely monitored every three months for a follow up duration of at least 12 months. All scans of the enrolled patients were acquired at the beginning of this study; thereafter patients were eligible to enter a 12-month follow-up period to evaluate the treatment responsiveness. At the end of the 12-month follow-up period, all the patients were classified into drug-responsive and drug-refractory groups. Refractory epilepsy was defined as ongoing seizures in the presence of two or more adequate antiepileptic drugs. The responsive group included the responders, defined as patients with a reduction of >50% of seizures, and the seizure-free patients, defined as disappearance of reported seizures after AED treatment.

A comparison group was selected by recruiting age- and sex-matched right-handed healthy controls (HCs) from the same regional population. Exclusion criteria were as follows: (1) any chronic medical disorder, (2) any convulsive episodes or a family history of epilepsy.

### Image acquisition

MRI scanning was performed on a 3.0 T Siemens Tim Trio MRI (Erlangen, Germany). The resting-state fMRI images were acquired using a gradient-echo-planer imaging (EPI) sequence, the scan parameters were as follows: TR = 2000 ms, TE = 35 ms, flip angle FA = 68°, slice thickness = 3 mm (no slice gap), matrix size = 64 × 64, FOV = 208 × 208 mm^2^, time point = 220, and acquisition time = 7.26 min, resulting in a voxel size of 3.3 × 3.3 × 3.0 mm^3^. Subjects were instructed to close their eyes, remain still, and relax, without falling asleep during scanning.

### Data preprocessing

Preprocessing of fMRI data was conducted using the Data Processing & Analysis for Brain imaging (DPABI)^33^ and SPM12 (statistical parametric mapping, http://wwwfil.ion.ucl.ac.uk/spm). Age and sex were entered into all group comparisons as nuisance covariates. We discarded the first 10 images to allow for magnetization equilibrium. For each participant, the remaining 210 EPI images were subjected to slice time correction, realigned motion (data were excluded if head motion exceeded 2 mm and 2°). Then, images were spatially normalized to the Montreal Neurologic Institute space and resampled to 3 × 3 × 3 mm^3^. Next, several spurious variances (24 head motion parameters, global signals, ventricular signals, and white matter signals) were regressed out using multiple linear regression analysis, and smoothed with 8 mm full-width at half-maximum (FWHM) Gaussian kernel. Subsequently, the functional images were trended and temporal band-pass filtered between 0.01 Hz and 0.08 Hz.

### ALFF calculation

Using the smoothed images, the average amplitude of low frequency fluctuation (ALFF) was calculated using REST software (http://www.restfmri.net/forum) running under Matlab (Mathworks, 2010 release). The time series of each voxel was firstly transformed to a frequency domain, and then the averaged square root of the power across 0.01–0.08 Hz was determined as the ALFF. The ALFF of each voxel was then divided by the global mean ALFF value of the individual to standardize data across subjects.

### Statistical analysis

We examined the differences in demographic information and clinical characteristics by performing statistical comparisons according to different variable types by Student *t* test, chi-squared test or ANOVA, using SPSS 20.0 software (SPSS, Chicago, IL, USA). Statistical significance was set at two-tailed P < 0.05.

Using the full factorial model in SPM12, we conducted one-way ANOVA to analysis the difference between the three groups of interest on ALFF. Age and sex were entered into all group comparisons as nuisance covariates. We set the statistical level at P < 0.05 and corrected for multiple comparisons with false discovery rate (FDR) correction. Next, we conducted post hoc analyses in GraphPad Prism to further identify definite ALFF alterations in a pairwise pattern amongst the responsive group, the refractory group, and the HC group. In each procedure of the group analysis, we added age, and gender as covariates.

To assess the relationship between epilepsy durations and ALFF value extracted in the patients’ group, we performed two-tailed Pearson correlation analyses using SPSS 20.0 (SPSS, Chicago, IL, USA). And a sensitivity sub-analysis of excluding outliers was also performed. P < 0.05 was used for analysis.

## Supplementary information

Supplementary Table 1

## Data Availability

The datasets generated during and/or analyzed during the current study are available from the corresponding author on reasonable request.

## References

[1] Blumcke I (2017). Histopathological Findings in Brain Tissue Obtained during Epilepsy Surgery. The New England journal of medicine.

[2] Kwan P, Brodie MJ (2000). Early identification of refractory epilepsy. The New England journal of medicine.

[3] Tailby C, Kowalczyk MA, Jackson GD (2018). Cognitive impairment in epilepsy: the role of reduced network flexibility. Annals of clinical and translational neurology.

[4] Manford M, Hart YM, Sander JW, Shorvon SD (1992). National General Practice Study of Epilepsy (NGPSE): partial seizure patterns in a general population. Neurology.

[5] Bonini F (2014). Frontal lobe seizures: from clinical semiology to localization. Epilepsia.

[6] Beleza P, Pinho J (2011). Frontal lobe epilepsy. Journal of clinical neuroscience: official journal of the Neurosurgical Society of Australasia.

[7] Hu Y, Jiang L, Yang Z (2012). Video-EEG monitoring differences in children with frontal and temporal onset seizures. The International journal of neuroscience.

[8] Rasmussen T (1991). Tailoring of cortical excisions for frontal lobe epilepsy. The Canadian journal of neurological sciences. Le journal canadien des sciences neurologiques.

[9] Law N, Smith ML, Widjaja E (2018). Thalamocortical Connections and Executive Function in Pediatric Temporal and Frontal Lobe Epilepsy. AJNR. American journal of neuroradiology.

[10] Klugah-Brown Benjamin, Luo Cheng, He Hui, Jiang Sisi, Armah Gabriel Kofi, Wu Yu, Li Jianfu, Yin Wenjie, Yao Dezhong (2018). Altered Dynamic Functional Network Connectivity in Frontal Lobe Epilepsy. Brain Topography.

[11] Evangelisti S (2018). Brain functional connectivity in sleep-related hypermotor epilepsy. NeuroImage: Clinical.

[12] Widjaja E, Zamyadi M, Raybaud C, Snead OC, Smith ML (2013). Abnormal functional network connectivity among resting-state networks in children with frontal lobe epilepsy. AJNR. American journal of neuroradiology.

[13] Zang YF (2007). Altered baseline brain activity in children with ADHD revealed by resting-state functional MRI. *Brain &*. development.

[14] Cao X (2014). Altered intrinsic connectivity networks in frontal lobe epilepsy: a resting-state fMRI study. Computational and mathematical methods in medicine.

[15] Etkin A, Egner T, Kalisch R (2011). Emotional processing in anterior cingulate and medial prefrontal cortex. Trends in cognitive sciences.

[16] Robinson LF (2017). The Temporal Instability of Resting State Network Connectivity in Intractable Epilepsy. Human brain mapping.

[17] Anderson SW, Bechara A, Damasio H, Tranel D, Damasio AR (1999). Impairment of social and moral behavior related to early damage in human prefrontal cortex. Nature neuroscience.

[18] Blair RJR, Cipolotti L (2000). Impaired social response reversalA case of ‘acquired sociopathy’. Brain: a journal of neurology.

[19] Zhang Z (2010). Altered spontaneous neuronal activity of the default-mode network in mesial temporal lobe epilepsy. Brain research.

[20] Braakman HM (2011). Cognitive and behavioral complications of frontal lobe epilepsy in children: a review of the literature. Epilepsia.

[21] Wang Z (2011). Altered resting state networks in epileptic patients with generalized tonic-clonic seizures. Brain research.

[22] Roland JL (2017). On the role of the corpus callosum in interhemispheric functional connectivity in humans. Proceedings of the National Academy of Sciences of the United States of America.

[23] Gotman J (2005). Generalized epileptic discharges show thalamocortical activation and suspension of the default state of the brain. Proceedings of the National Academy of Sciences of the United States of America.

[24] Rasmussen T (1991). Surgery for central, parietal and occipital epilepsy. The Canadian journal of neurological sciences. Le journal canadien des sciences neurologiques.

[25] Tellez-Zenteno JF, Dhar R, Wiebe S (2005). Long-term seizure outcomes following epilepsy surgery: a systematic review and meta-analysis. Brain: a journal of neurology.

[26] Nagai Y (2018). Epileptic Seizures are Reduced by Autonomic Biofeedback Therapy Through Enhancement of Fronto-limbic Connectivity: A Controlled Trial and Neuroimaging Study. EBioMedicine.

[27] Trebuchon A, Bartolomei F, McGonigal A, Laguitton V, Chauvel P (2013). Reversible antisocial behavior in ventromedial prefrontal lobe epilepsy. Epilepsy & behavior: E&B.

[28] Woodward KE, Gaxiola-Valdez I, Goodyear BG, Federico P (2014). Frontal lobe epilepsy alters functional connections within the brain’s motor network: a resting-state fMRI study. Brain connectivity.

[29] Critchley HD, Harrison NA (2013). Visceral influences on brain and behavior. Neuron.

[30] Vaessen MJ (2014). Functional and structural network impairment in childhood frontal lobe epilepsy. PloS one.

[31] Vaessen MJ (2013). Abnormal modular organization of functional networks in cognitively impaired children with frontal lobe epilepsy. Cerebral cortex (New York, N.Y.: 1991).

[32] Hirose G (2013). An overview of epilepsy: its history, classification, pathophysiology and management. Brain and nerve=Shinkei kenkyu no shinpo.

[33] Yan CG, Wang XD, Zuo XN, Zang YF (2016). DPABI: Data Processing & Analysis for (Resting-State) Brain Imaging. Neuroinformatics.

