# Supplementary material for: Altered intrinsic brain activity associated with outcome in frontal lobe epilepsy
Source: Sci Rep. 2019 Jun 20;9:8989. doi: 10.1038/s41598-019-45413-7 (PMC6586796; doi:10.1038/s41598-019-45413-7)
Supplement: Supplementary file 1 — Supplementary Table 1 [file 41598_2019_45413_MOESM1_ESM.docx]

**Altered intrinsic brain activity associated with outcome in frontal lobe epilepsy**

***Xintong Wu^1#^, Wenyu Liu^1#^, Weina Wang^2^, Hui Gao^1^, Nanya Hao^1^, Qiang Yue^2*^, Qiyong Gong^2^, Dong Zhou^1*^***

1.Departments of Neurology, Huaxi MR Research Center (HMRRC), West China Hospital, Sichuan University, No. 37 GuoXue Alley, Chengdu, 610041, China;

2. Departments of Radiology, West China Hospital, Sichuan University, No. 37 GuoXue Alley, Chengdu, 610041, China;

*Correspondence to: Qiang Yue, Electronic address: scu_yq@163.com; and Dong Zhou, zhoudong66@yahoo.de;

^#^ These two authors contribute equally.

**Supplementary Table 1 The information of patients with FLE in the study**

| Group | No. | Age | Seizure onset | | Dur. | | Seizure  type | Baseline freq./year | | End  freq. /year | | | B  medication | E  medication | MMSE | | Interictal EEG | Ictal EEG | | PET |
| --- | --- | --- | --- | --- | --- | --- | --- | --- | --- | --- | --- | --- | --- | --- | --- | --- | --- | --- | --- | --- |
| Resp. | 1 | 49 | 42 | 7 | | CPS,sGTCS | | | 10 | | 2 | OXC, LEV | | OXC, LEV | | 25 | B F spikes | | L F | normal |
|  | 2 | 27 | 21 | 6 | | SPS,sGTCS | | | 7 | | 4 | LTG, LEV | | LTG, LEV | | 28 | F spikes, L>R | | L F | L F hypometabolism |
|  | 3 | 14 | 3 | 11 | | SPS,sGTCS | | | 8 | | 3 | OXC, LEV | | OXC, LEV | | 26 | B slow | | R FP | normal |
|  | 4 | 63 | 53 | 10 | | CPS,sGTCS | | | 11 | | 6 | LEV | | LEV | | 25 | F, R>L spikes | | - | - |
|  | 5 | 35 | 15 | 20 | | GTCS | | | 4 | | 1 | CBZ | | CBZ | | 27 | normal | | B F | normal |
|  | 6 | 27 | 10 | 17 | | CPS,GTCS | | | 8 | | 4 | CBZ, LEV | | CBZ, LEV | | 29 | L F spikes | | - | - |
|  | 7 | 25 | 22 | 3 | | CPS,sGTCS | | | 9 | | 5 | VPA, LEV | | VPA, LEV | | 26 | B F slow | | R F | - |
|  | 8 | 37 | 25 | 12 | | CPS,GTCS | | | 6 | | 3 | TPM, LEV | | TPM, LEV | | 25 | R F spikes | | R F | - |
|  | 9 | 14 | 9 | 5 | | CPS,sGTCS | | | 16 | | 7 | OXC, LEV | | OXC, LEV | | 28 | B spikes, L>R | | L FT | L F hypometabolism |
|  | 10 | 17 | 13 | 4 | | GTCS | | | 6 | | 1 | VPA, LEV | | VPA, LEV | | 25 | B spikes | | L F | - |
|  | 11 | 35 | 18 | 17 | | CPS,GTCS | | | 7 | | 2 | OXC | | OXC | | 24 | L FT spikes | | - | - |
|  | 12 | 13 | 3 | 10 | | GTCS | | | 9 | | 4 | VPA, LEV | | VPA, LEV | | 25 | B slow | | R F | R FT hypometabolism |
|  | 13 | 13 | 8 | 5 | | GTCS | | | 5 | | 0 | LEV | | LEV | | 27 | R F spikes | | R F | - |
|  | 14 | 26 | 17 | 9 | | CPS,GTCS | | | 22 | | 8 | OXC, LEV | | OXC, LEV | | 27 | R F spikes | | R O | R FO hypometabolism |
|  | 15 | 18 | 12 | 6 | | GTCS | | | 5 | | 1 | OXC | | OXC | | 25 | L F spikes | | L FT | - |
|  | 16 | 34 | 28 | 6 | | GTCS | | | 9 | | 2 | OXC, LEV | | OXC, LEV | | 25 | L T spikes | | L FT | - |
|  | 17 | 46 | 45 | 1 | | CPS,GTCS | | | 7 | | 3 | CBZ | | CBZ | | 28 | B FT spikes | | R F | - |
|  | 18 | 17 | 12 | 5 | | GTCS | | | 9 | | 5 | VPA,LEV | | VPA,LEV | | 27 | L FPT spikes | | - | - |
|  | 19 | 32 | 27 | 5 | | GTCS | | | 7 | | 3 | VPA,LEV | | VPA,LEV | | 28 | R F spike-slow | | R F | - |
|  | 20 | 29 | 19 | 10 | | CPS,GTCS | | | 15 | | 4 | OXC,LEV | | OXC,LEV | | 27 | B FT spike-slow | | L F | L F hypometabolism |
|  | 21 | 26 | 19 | 7 | | sGTCS | | | 7 | | 3 | VPA, LEV | | VPA, LEV | | 25 | B FT spikes | | - | - |
|  | 22 | 57 | 54 | 3 | | SPS | | | 12 | | 6 | OXC, LEV | | OXC, LEV | | 26 | L FT spike-slow | | L FT | - |
|  | 23 | 49 | 38 | 11 | | GTCS | | | 10 | | 3 | LTG | | LTG | | 25 | normal | | B F | - |
|  | 24 | 33 | 28 | 5 | | CPS,GTCS | | | 5 | | 2 | OXC | | OXC | | 29 | B FT spikes | | B F | normal |
|  | 25 | 30 | 17 | 13 | | CPS | | | 9 | | 3 | OXC,LEV | | OXC,LEV | | 24 | R FTO L F spikes | | R F | - |
| Refr. | 26 | 42 | 24 | 18 | | CPS,sGTCS | | | 12 | | 10 | VPA, LEV | | VPA, LEV | | 28 | B spikes | | R FT | R FT hypometabolism |
|  | 27 | 25 | 21 | 4 | | CPS | | | 10 | | 7 | CBZ | | CBZ, LEV | | 27 | B slow | | B F | - |
|  | 28 | 51 | 36 | 15 | | CPS,sGTCS | | | 6 | | 5 | TPM, LEV | | TPM, LEV | | 25 | L F spikes | | - | - |
|  | 29 | 27 | 23 | 4 | | sGTCS | | | 5 | | 8 | OXC | | OXC, LEV | | 27 | B spikes, L>R | | - | - |
|  | 30 | 17 | 9 | 8 | | CPS | | | 24 | | 22 | CBZ, VPA | | CBZ, VPA, LTG | | 25 | B spike, R>L | | R FT | R FT hypometabolism |
|  | 31 | 17 | 13 | 4 | | CPS,sGTCS | | | 8 | | 7 | VPA, CBZ | | VPA, CBZ | | 27 | R FTO spikes | | R FT | R F hypometabolism |
|  | 32 | 27 | 18 | 9 | | GTCS | | | 4 | | 3 | VPA, LEV | | VPA, LEV | | 26 | B slow | | R F | - |
|  | 33 | 30 | 10 | 20 | | CPS,GTCS | | | 6 | | 6 | OXC, LTG | | OXC, LTG | | 27 | R>L slows | | R F | - |
|  | 34 | 18 | 10 | 8 | | CPS,sGTCS | | | 8 | | 5 | LEV | | OXC, LEV | | 25 | B spikes | | R FP | - |
|  | 35 | 25 | 20 | 5 | | GTCS | | | 12 | | 8 | OXC, TPM | | OXC, TPM | | 26 | B spikes | | B F | - |
|  | 36 | 27 | 3 | 24 | | CPS,sGTCS | | | 9 | | 6 | VPA, LEV | | VPA, LEV | | 29 | L F spikes | | - | - |
|  | 37 | 42 | 4 | 38 | | CPS,sGTCS | | | 13 | | 10 | CBZ, VPA | | CBZ, VPA | | 24 | L FTO spikes | | L F | L F hypometabolism |
|  | 38 | 30 | 26 | 4 | | GTCS | | | 8 | | 6 | CBZ, VPA | | CBZ, VPA | | 30 | normal | | L F | - |
|  | 39 | 26 | 24 | 2 | | SPS | | | 10 | | 8 | OXC, LEV | | OXC, LEV | | 27 | R FT spikes | | R F | R PT hypometabolism |
|  | 40 | 18 | 10 | 8 | | GTCS | | | 7 | | 5 | VPA | | VPA | | 28 | L F spikes | | - | - |
|  | 41 | 34 | 27 | 7 | | CPS,sGTCS | | | 12 | | 11 | PHT, CBZ | | PHT, CBZ | | 25 | L FT spikes | | L FT | - |
|  | 42 | 44 | 24 | 20 | | SPS,sGTCS | | | 8 | | 5 | TPM, LEV | | TPM, LEV | | 27 | B spikes | | L FT | R T hypometabolism |
|  | 43 | 22 | 17 | 5 | | CPS,GTCS | | | 11 | | 8 | OXC | | OXC, LEV | | 26 | L>R slows | | L F | R F hypometabolism |
|  | 44 | 29 | 13 | 16 | | GTCS | | | 5 | | 4 | LTG, LEV | | LTG, LEV | | 26 | L FT spike-slow | | - | - |
|  | 45 | 18 | 14 | 4 | | CPS | | | 6 | | 5 | TPM | | TPM, CBZ | | 28 | L FTP spikes | | L F | - |
|  | 46 | 26 | 19 | 7 | | CPS,GTCS | | | 9 | | 9 | CBZ, VPA | | CBZ, VPA | | 28 | B FT spikes | | R F | - |
|  | 47 | 24 | 12 | 12 | | CPS,sGTCS | | | 10 | | 7 | OXC, LEV | | OXC, LEV | | 26 | R F spikes | | - | - |
|  | 48 | 21 | 6 | 15 | | GTCS | | | 6 | | 9 | OXC, LTG | | OXC, LTG | | 29 | B FT spikes | | L FT | - |
|  | 49 | 30 | 20 | 10 | | CPS,sGTCS | | | 12 | | 10 | OXC, LEV | | OXC, LEV | | 27 | B spike, R>L | | R FT | R FT hypometabolism |
|  | 50 | 25 | 3 | 22 | | CPS,sGTCS | | | 15 | | 11 | CBZ, VPA | | CBZ, VPA | | 27 | B spikes, L>R | | L F | B FT hypometabolism |

Abbreviations:

Dur. Duration; freq. frequency; B bilateral; F frontal; T temporal; P parietal; O occipital; L left; R right; CPS complex partial seizure; GTCS Generalized tonic-clonic seizure; sGTCS secondarily Generalized tonic-clonic seizure; OXC oxcarbazepine; CBZ carbamazepine; LEV Levetiracetam; LTG Lamotrigine; VPA valproate ; PHT phenytoin; MMSE Mini-mental state examination; EEG electroencephalography; PET  positron emission tomography; Resp. responsive group; Refr. refractory group; FLE frontal lobe epilepsy.

The Information including patients’ age, age of seizure onset, duration of epilepsy, seizure type, MMSE, EEG and PET was collected at baseline. In addition, seizure frequency and used medications were recorded both at baseline and 12-month follow up period. At the end of the study period, patients from no. 1 to 25 were classified into the responsive group, and the rest (26-50) were classified into the refractory group.
